# Top‐down versus bottom‐up attention differentially modulate frontal–parietal connectivity

**DOI:** 10.1002/hbm.24850

**Published:** 2019-11-06

**Authors:** Jake T. Bowling, Karl J. Friston, Joseph B. Hopfinger

**Affiliations:** ^1^ School of Medicine University of North Carolina at Chapel Hill Chapel Hill North Carolina; ^2^ Wellcome Trust Centre for Neuroimaging University College London London UK; ^3^ Department of Psychology and Neuroscience University of North Carolina at Chapel Hill Chapel Hill North Carolina; ^4^ Biomedical Research Imaging Center University of North Carolina at Chapel Hill Chapel Hill North Carolina

**Keywords:** dynamic causal modeling, dorsal attention network, endogenous, exogenous, fMRI, involuntary attention, voluntary attention

## Abstract

The moment‐to‐moment focus of our mind's eye results from a complex interplay of voluntary and involuntary influences on attention. Previous neuroimaging studies suggest that the brain networks of voluntary versus involuntary attention can be segregated into a frontal‐versus‐parietal or a dorsal‐versus‐ventral partition—although recent work suggests that the dorsal network may be involved in both bottom‐up and top‐down attention. Research with nonhuman primates has provided evidence that a key distinction between top‐down and bottom‐up attention may be the *direction* of connectivity between frontal and parietal areas. Whereas typical fMRI connectivity analyses cannot disambiguate the direction of connections, dynamic causal modeling (DCM) can model directionality. Using DCM, we provide new evidence that directed connections within the dorsal attention network are differentially modulated for voluntary versus involuntary attention. These results suggest that the intraparietal sulcus exerts a baseline inhibitory effect on the frontal eye fields that is strengthened during exogenous orienting and attenuated during endogenous orienting. Furthermore, the attenuation from endogenous attention occurs even with salient peripheral cues when those cues are known to be counter predictive. Thus, directed connectivity between frontal and parietal regions of the dorsal attention network is highly influenced by the type of attention that is engaged.

## 1 INTRODUCTION

Accurate perception and action depend on the ability of attention systems to focus processing resources on (i.e., select) the most salient stimuli in the environment. A wealth of experimental evidence shows that selective spatial attention leads to faster and more accurate responses to stimuli at an attended location (see Pashler, 1998). Neuroimaging studies in humans and lesion analysis in neurological patients and nonhuman primates have provided evidence that the orienting of attention is supported by a widespread network, including the posterior parietal cortex, temporoparietal junction (TPJ), superior temporal sulcus, and dorsal regions of the frontal cortex (Corbetta et al., 1998; Corbetta, Kincade, Ollinger, McAvoy, & Shulman, 2000; Corbetta, Miezin, Shulman, & Petersen, 1993; Friedrich, Egly, Rafal, & Beck, 1998; Gitelman et al., 1999; Henik, Rafal, & Rhodes, 1994; Hopfinger, Buonocore, & Mangun, 2000; Kim et al., 1999; Nobre et al., 1997; Posner & Cohen, 1984; Watson, Valenstein, Day, & Heilman, 1994).

It is generally agreed that attentional orienting can be accomplished either voluntarily, as an effortful act, or reflexively, via capture by salient sensory events in the sensorium. Crucially, it is the interplay of voluntary and involuntary influences that determine the moment‐to‐moment focus of our mind's eye. Previous research has identified similarities—and key differences—between these types of attention. Whereas both types of attention appear to rely upon frontal–parietal control systems, and both can modulate processing in sensory processing regions, there are notable differences. For example, behavioral studies have shown differences between these types of orienting (Jonides, 1981; Müller & Rabbitt, 1989; Posner & Cohen, 1984; Prinzmetal, Zvinyatskovskiy, Gutierrez, & Dilem, 2009; Warner, Juola, & Koshino, 1990; Wright & Richard, 2000; Yantis, 1993). Involuntary attention—often referred to as exogenous attention because it is thought to be triggered by external stimuli—can be engaged more rapidly and is more resistant to interference than is the type of attention referred to as voluntary, or endogenous (from within; initiated by internal goals) attention (e.g., Jonides, 1981; Müller & Rabbitt, 1989). Involuntary and voluntary attention have also been shown to unfold over different time courses (e.g., Cheal & Lyon, 1991; Müller & Rabbitt, 1989; Posner & Cohen, 1984). Involuntary orienting to a location results in faster and more accurate responses to items at that location, but only for a few hundred millisecond. At intervals longer than ~300 ms (between an involuntary cue and target), responses are actually slower at the cued location—a phenomenon termed inhibition of return (Posner & Cohen, 1984). Voluntary attention, in contrast, is somewhat slower to engage, but results in a sustained advantage at the attended location.

Results from neuroscience studies have also shown dissociations between these types of attention (Hodsoll, Mevorach, & Humphreys, 2009; Hopfinger, Parsons, & Fröhlich, 2017; Hopfinger & West, 2006; Rossi, Bichot, Desimone, & Ungerleider, 2007). Neuropsychological evidence suggests that partially or wholly separate neural mechanisms support voluntary versus involuntary orienting (Rafal, 1996), and recent neuroimaging studies have found some differences between the control networks supporting these types of orienting (Hahn, Ross, & Stein, 2006; Mayer, Dorflinger, Rao, & Seidenberg, 2004; Mort et al., 2003; Rosen et al., 1999). Studies have suggested that voluntary attention is associated with more dorsal regions of the frontal and parietal lobes, whereas involuntary attention is associated with more ventral regions, including the temporoparietal junction (Corbetta & Shulman, 2002).

Despite the growing evidence that the control of these types of orienting may be distinct, it is less clear whether these systems orient the same “spotlight” of attention. Specifically, it is often assumed that—regardless of the means of orienting attention (voluntarily pushed there or involuntary captured there)—spatial attention does the same thing, once it is focused on a location. However, a number of recent findings suggest that voluntary and involuntary attention may actually be orienting two different “spotlights.” Briand (1998) and Briand and Klein (1987) have shown that voluntary and involuntary orienting have qualitatively distinct effects on processing. Specifically, they find an interaction between attention type (voluntary vs. involuntary) and search type (conjunction vs. feature), with only involuntary attention producing a significant difference between these types of search. Berger, Henik, and Rafal (2005) also provide evidence for the separation of these types of attention, showing that when both types of attention are engaged on the same trials, they have independent effects on response time. Prinzmetal, McCool, and Park (2005) provide additional evidence that voluntary and involuntary orienting are psychophysically distinct, finding that involuntary attention affects reaction times, but not accuracy, whereas voluntary attention significantly affects both. These findings do not yet converge on a simple explanation for the differences between voluntary and involuntary attention; however, they do provide evidence that voluntary and involuntary attention involve different mechanisms.

Whereas neuroimaging evidence has shown largely overlapping regions implicated in voluntary and involuntary attention, crucial insights into the mechanisms of these types of attention come from single‐unit recordings from macaque monkeys. Specifically, Buschman and Miller (2007) demonstrated critical differences in the temporal order of frontal and parietal activities between endogenous and exogenous attention. In that study, frontal activity preceded parietal activity when the animal was orienting endogenously in response to an informative cue. However, these same brain regions showed the opposite temporal pattern—with parietal activation leading frontal activity—when orienting was triggered in an exogenous manner by salient, uninformative, stimuli. Despite this definitive finding, functional neuroimaging results have typically found highly similar patterns of activity across these two types; primarily showing activation in a bilateral frontoparietal network (Corbetta et al., 1993; Kim et al., 1999; Kincade, Abrams, Astafiev, Shulman, & Corbetta, 2005; Nobre et al., 1997; Peelen, Heslenfeld, & Theeuwes, 2004), even in the complete absence of any cue stimulus (Hopfinger, Camblin, & Parks, 2010). The sluggish nature of the hemodynamic response may obscure important timing differences within the frontoparietal network when traditional fMRI analyses are used. The present study aims to advance previous work by measuring directed connectivity in the dorsal frontoparietal network for endogenous and exogenous attention, using an optimized fMRI protocol and dynamic causal modeling (DCM), an established method for investigating directed connectivity in the brain (Friston, Harrison, & Penny, 2003). Based on Buschman and Miller's (2007) results, we specifically asked if the directed connectivity between dorsal frontal and parietal attentional control regions is differentially modified by the type of attention being engaged.

## 2 METHODS

This experiment uses a dataset that has been described in a previous, recent publication (Meyer, Du, Parks, & Hopfinger, 2018). Additional details regarding participants, tasks, protocols, and data preprocessing can be found there.

### 2.1 Participants

Twenty healthy young adults participated and were paid $20/hr. Exclusion criteria included a history of neurological or psychiatric illness, implanted metal, pregnancy, color blindness, and uncorrected abnormal vision. All procedures were approved by the Institutional Review Board at the University of North Carolina at Chapel Hill. All participants provided informed consent in writing. One participant was unable to complete all the runs of all conditions in the allotted time; therefore, the present analysis includes 19 participants (ages 19–32, 10 females).

### 2.2 Tasks

**Figure 1:**
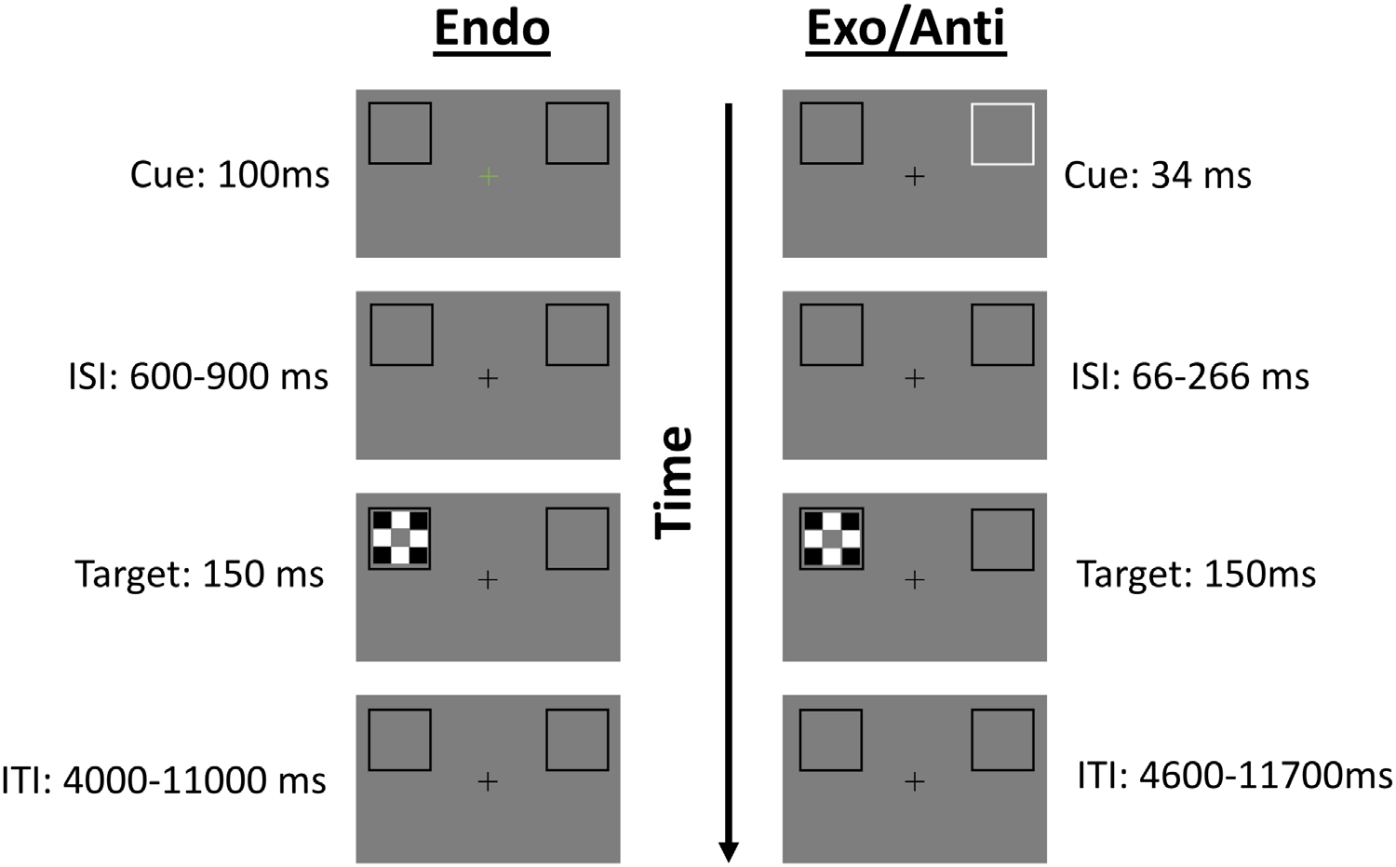
Trial sequence and stimuli. Left column: Endogenous (“Endo”) cue condition. The cue was a color change of the fixation cross from black to either red or green (indicating which side the target was likely to appear). Then, 25% of the trials did not contain any target stimulus (“catch” trials). In the other 75% of trials, targets appeared in the expected location 83% of the time and in the unexpected location 17% of the time. Right column: Exogenous (“Exo”) and Anti‐predictive (“Anti”) conditions both used the same physical stimuli and timing. As in the endogenous condition, 25% of trials were catch trials, with no target. For target‐present trials in the Exo condition, the target occurred equally often at the cued and uncued locations (i.e., the cue was not predictive of target location). For target‐present trials in the anti‐condition, the target occurred at the expected location (opposite side from the cue stimulus) 83% of the time and at the unexpected location (same side as the cue) 17% of the time

Participants viewed images on a translucent screen through an angled mirror attached to the head coil. Commercial software (Neurobehavioral Systems, San Francisco, CA) was used to present stimuli and record responses and reaction times. Before completing experimental runs, all participants completed a behavioral training session to ensure high performance accuracy. There were three different attention conditions; endogenous, exogenous, and anti‐predictive exogenous. Conditions were blocked such that participants completed only one condition type per run. Each run consisted of 64 trials. Participants completed two runs of each attention condition for a total of six functional runs (384 trials) per participant. The order of conditions was counterbalanced across participants. An illustration of the trial paradigm is shown in Figure 1. Each trial began with a 600–700 ms presentation of a black fixation cross and two black outline boxes (3.2 × 3.2 in.) against a dark gray background. The boxes appeared above midline and were 5 in. to the left or right of the central fixation cross. Participants were instructed to keep their eyes fixated on the central cross.

In the endogenous cueing condition, a color change of the fixation cross signaled participants to engage voluntary attention. After 600–700 ms, the central cross flashed either green (50%) or red (50%) for 100 ms. Participants were instructed that the color of the cross indicated the most likely (75% valid) location of the upcoming target on that trial. For half of the participants, a green cross indicated that the target was more likely to appear on the left side, and a red cross indicated the target was more likely on the right side; the color‐location predictability was reversed for the other half of participants. All participants were instructed truthfully about the color‐location mapping condition they were in. After the cue, there was an interstimulus interval (ISI) of 600–900 ms during which participants *covertly* shifted attention to the anticipated target location. Then, the target, a black‐and‐white checkerboard, appeared in one of the two peripheral boxes for 150 ms. The center of the checkerboard square was a solid gray square for 50% of trials and alternated between white and black for the other 50%. Participants were asked to report as quickly and accurately as possible whether the center was gray or not by pressing a key. Intertrial intervals (ITIs) ranged from 4 to 11 s. The target did not appear at all for 25% of the total trials (“catch” trials; 16/64 trials of each run). Of the 48/64 trials per run in which the target did appear, in 40/48, the cue color predicted the location accurately. Although participants were not formally tested for color blindness, all participants were asked if they were colorblind during screening and only subjects reporting no colorblindness were enrolled in the study. In addition, all subjects reported being able to differentiate the red cues versus the green cues during training before the MRI sessions.

In the exogenous condition, involuntary attention was captured using a salient peripheral flash. As in the endogenous condition, trials began with a 600–700 ms preview display of a fixation cross. Next, one of the two black boxes flashed white for 34 ms. Participants were instructed (truthfully) that this cue was *uninformative* about the upcoming target location. After the flash, there was an ISI of 66–266 ms before the target appeared for 150 ms. The checkerboard target and task goal were the same as in the endogenous condition. The ITI varied from 4,600 to 11,700 ms to balance our overall run length across conditions. Again, 25% of trials were catch trials. For the 48/64 trials with a target, in 24/48 the cue and target appeared in the same location, and in the other 24/48, the target appeared opposite the cue.

In the anti‐predictive condition, physical stimuli were identical to the exogenous condition; a 34 ms salient white flash followed by a target (75%) or no target (25%; “catch trials”). However, for this condition, the cue (flash) was *predictive* of the target location being *opposite* the cue, and participants were instructed as such. In 83% of target‐present trials, the target appeared opposite the cue, and in 17% of target‐present trials, the target appeared in the same location as the cue.

### 2.3 MRI scanning protocol

Images were collected using a 3 T Siemens Trio Total imaging matrix MRI system at the University of North Carolina Biomedical Research Imaging Center. Partial brain imaging focused on the cortex at the level of the dorsal frontoparietal network, allowing rapid image acquisition. A structural scan was acquired for each participant before the experiment (T1, TR = 1,900 ms, TE = 2.32 ms, flip angle = 9, FOV = 230 mm, 192 slices, 0.9 × 0.9 × 0.9 mm^3^ resolution). Functional images included 10 transverse slices (4 × 4 × 6 mm^3^ resolution) collected interleaved inferior to superior. Images were acquired using a T2‐weighted echo‐planar imaging (EPI) sequence (TR = 500 ms, TE = 27 ms, flip angle = 90°), and the first two scans were discarded to allow for magnetic field stabilization. Participants completed two runs of each condition for a total of six runs (7.3 min each, 43.8 min total).

### 2.4 Preprocessing

MRI data were processed and analyzed using SPM12 (Wellcome Department of Imaging Neuroscience, University College London, UK). Preprocessing steps included slice‐time correction, spatial realignment, and spatial normalization, using the mean image constructed at the realignment stage to determine parameters to normalize the EPI datasets into MNI space using the EPI template). Data were then smoothed with an 8 mm (FWHM) isotropic kernel.

### 2.5 GLM, regions of interest, and time series extraction

The fMRI timeseries from all sessions from a given subject were concatenated, and the three attention conditions were modeled as boxcar functions in the usual way (using a general linear model). In the GLM, each session included two conditions: catch (target‐absent) trials and target‐present trials. There were an additional eight nuisance regressors in the model: six movement parameters of the rigid body realignment and two physiologic regressors representing cerebrospinal fluid (CSF) and white matter (WM) signal. Coordinates for the two physiologic regressors were identified separately: CSF from the Harvard‐Oxford Atlas (Harvard Center for Morphometric Analysis) (x,y,z: −4,6,12) and WM from the Johns Hopkins University White Matter Atlas (Mori, Wakana, Nagae‐Poetscher, & van Zijl, 2005) (x,y,z: −20,26,28). Physiologic regressors were generated in MarsBar (Brett, Anton, Valabregue, & Poline, 2002) using 1.5 mm radius spheres centered on these respective coordinates. Event onset times and nuisance regressor vectors were concatenated across runs. The GLM contained an additional six regressors generated by the concatenate function in SPM; these controlled for session (run) effects.

We then selected regions of interest (ROIs) for subsequent DCM. ROIs included the bilateral frontal eye fields (FEFs) and intraparietal sulci (IPS), in accordance with literature on the dorsal attention network (Buschman & Miller, 2007; Hopfinger et al., 2000; Meehan et al., 2017). For consistency with prior work on this dataset, our ROI‐identification procedure started from the group‐averaged coordinates reported in that previous work (Meyer et al., 2018). We performed a 6 mm small‐volume search centered on those group‐averaged coordinates, from which we identified a local maximum for each individual as the center of each of the four ROIs. A contrast for the effect of all conditions was used to identify these peak voxels. All participants had significantly active voxels within each ROI. Using a T‐contrast for all conditions, mean‐corrected (by an F‐contrast for effects of interests) time series from each participant were collected within 6 mm radius spherical volumes centered on each of the four ROIs with the first eigenvariate of voxels above a threshold of *p* < .001 (uncorrected).

### 2.6 Dynamic causal modeling

We used DCM12, as implemented in SPM12, to measure effective connectivity in the dorsal frontoparietal network. DCM is a Bayesian framework for inferring hidden neuronal states from observed measurements of neural activity (Friston et al., 2003; Stephan et al., 2010). In DCM for fMRI, bilinear differential equations describe how neural states change as a function of average (“fixed”) connections between regions (DCM.A matrix), bilinear or modulatory effects on these connections (DCM.B), and driving inputs (DCM.C) to regions themselves (Stephan, Weiskopf, Drysdale, Robinson, & Friston, 2007). An advantage of DCM—over other methods of connectivity analysis—is that, within a restricted hypothesis domain, its state‐space equation relates the temporal and spatial information encoded in fMRI data with user‐specified information about when and where the network is perturbed by external manipulations (e.g., task events). This allows causal inference on both the directed effect of one region on another and also on how that effect changes under experimental conditions. Our objective was to test particular hypotheses about where attentional set exerted its effects on directed hierarchical connectivity in the dorsal frontoparietal network. This represents an interesting challenge because different combinations of attentional effects can be expressed in different combinations of connections. Our particular questions related to differential effects on forward versus backward connectivity under endogenous and exogenous attention conditions.

With DCM, the first step is to construct a set of models that represent competing hypotheses about the connectivity architecture in the network of interest. We specified models in which attention could selectively modulate various combinations of connections (as encoded by the B matrix in DCM); where the underlying (average) connectivity is denoted by an adjacency A matrix. This means that there are as many B matrices as attentional effects. Our models or hypotheses were therefore defined in terms of the B matrices that specify where attentional effects are deployed. Our models included reciprocal connectivity between regions across‐hemisphere and within‐hemisphere. In order to limit the number of models, we did not include every possible connection between areas. In particular, we did not include heterotopic connections across hemispheres (e.g., left IPS to right FEF) because they are generally considered to be less plausible than a combination of homotopic connections and “U” fibers (Stephan, Tittgemeyer, Knosche, Moran, & Friston, 2009). To best isolate the effects of attentional processing—and distinguish our conditions—catch trials from each condition were specified as modulatory input on frontoparietal connections. This approach to examining attentional effects removes any confounding influence of target processing and response. All other (cue + target) trials entered the model as driving input to the bilateral FEF and IPS. This inclusive and conservative treatment of driving effects (at all regions) precludes any possible bias in terms of inferring attentional modulation in forward and backward connections. We created and tested 188 models per participant. This set included all combinations of models with different attentional effects on bilateral forward (IPS to FEF) and backward (FEF to IPS) connections (i.e., 64 models in which each condition could modulate bilateral forward, bilateral backward, all four, or no frontoparietal connections). To assess possible hemispheric asymmetry in attention effects, we included an additional 124 models that lacked one of the unilateral, unidirectional attentional modulatory effects, for all combinations of conditions (i.e., models in which each condition could modulate any three out of four frontoparietal connections).

### 2.7 Bayesian model selection and averaging

We adopted two approaches to testing our hypotheses. First, model evidence was pooled over subjects for each of the specified 188 models to identify the most plausible hypothesis or explanation for the data in terms of attentional modulation. Each parameter of a DCM is constrained by a prior distribution that reflects conservative assumptions of plausible values (Stephan et al., 2010). BMS compares the free energy approximation to the log evidence of a set of models; in other words, it selects the most likely model structure with regards to a balance between model complexity (i.e., number of parameters) and goodness of fit (i.e., accuracy). These model comparisons ask whether there is evidence for the existence of a particular attentional effect on various combinations of connections. We used a fixed‐effects (FFX) approach to model selection at the group level. With FFX, one assumes the optimal (condition‐specific) model architecture is shared by all individuals in the population, and that individual differences are at the level of effective connectivity *strengths*; this assumption is warranted when studying basic cognitive processes in a healthy adult single‐group population (Stephan et al., 2010). FFX BMS uses the group Bayes factor—the product of Bayes factors over *N* subjects—which encodes the probability that the data were generated by one model relative to others, under the assumption that all subjects share a single model.

### 2.8 Inference on parameters/statistical analysis

After selection of a winning model, we used Bayesian parameter averaging (BPA) to further examine differences between our conditions at the level of individual parameters. BPA computes an average of parameters for a single model over multiple participants. BPA is a Bayesian approach in that it calculates posteriors, not with regard to the probability of models but with the precision of individual parameter estimates (the inverse of the parameter posterior covariance matrix) (Penny, 2013). We then examined the posterior probability matrix (encoded in DCM.Pp) to test whether Bayesian parameter averages of attentional effects differed from a null hypothesis of zero. Next, we examined contrasts between endogenous and exogenous attention on frontoparietal connections to test against a null hypothesis of no difference (0 Hz), using the contrast of connections function in DCM.

We used this form of BPA—as opposed to random effects Bayesian Model Comparison or Parametric Empirical Bayes over subjects—because we were not interested in establishing the presence of attentional effects, or whether they were conserved over subjects: our question was whether there were quantitative asymmetries in attentional effects on forward and backward connections, assuming that they exist in all subjects. In this setting, BPA provides a very efficient estimate of (changes in) directed connectivity, by accumulating evidence over subjects.

## 3 RESULTS

### 3.1 Full volume, GLM random effects analysis

**Figure 2:**
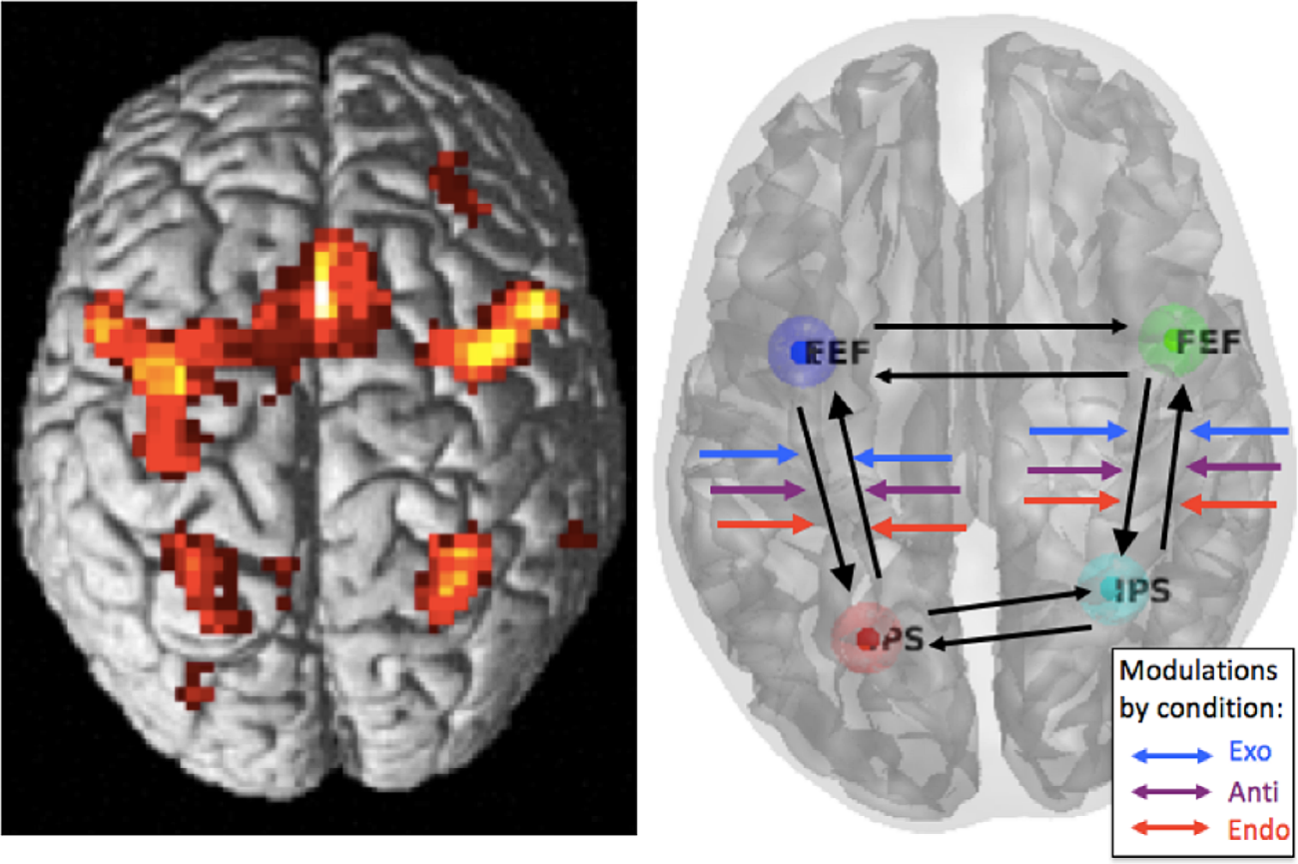
Left: Results from random effects analysis (*p* < .05 FWE) of catch trial (cue‐only) events for all conditions (Exo, Endo, and Anti). Right: Schematic of dynamic causal modeling (DCM) model space, showing the four regions used in these DCM analyses, intrinsic connections, and modulatory inputs. Not shown are the driving inputs (target‐present trials) which entered all four regions of interest (ROIs). The DCM analysis focused on how each condition (indicated by the blue, purple, and red arrows) modulated the FEF‐ > IPS and IPS‐ > FEF connectivity

Brain regions that responded significantly to cue stimuli are shown in Figure 2 (left panel). As expected, this whole volume analysis revealed robust activation throughout the dorsal frontoparietal network. Here, we focus on the FEF and IPS, regions previously found to be crucial for attention, as identified in our earlier report of this dataset (Meyer et al., 2018). The group‐level coordinates used for the current DCM analyses are presented in Table 1; these coordinates are the group average from taking each individual's location of maximal activation within the 6 mm region centered on our previous report of this dataset (Meyer et al., 2018). Figure 2 (right panel) illustrates the connections between these regions that are tested in the current analyses.

**Table 1:** Coordinates of ROIs in MNI space. Coordinates are group averaged over each individual's location of maximal activity (contrast for all conditions) within the 6 mm region centered on our previous results (Meyer et al., 2018). *T*‐scores also group averaged

ROI	*X*	*Y*	*Z*	*T*‐value (mean)
Left FEF	−37	−7	58	13.26
Right FEF	46	1	42	9.74
Left IPS	−24	−63	48	12.38
Right IPS	30	−56	46	11.21

Abbreviations: FEF, frontal eye field; IPS, intraparietal sulcus; ROI, region of interest.

### 3.2 DCM: Driving inputs

**Figure 3:**
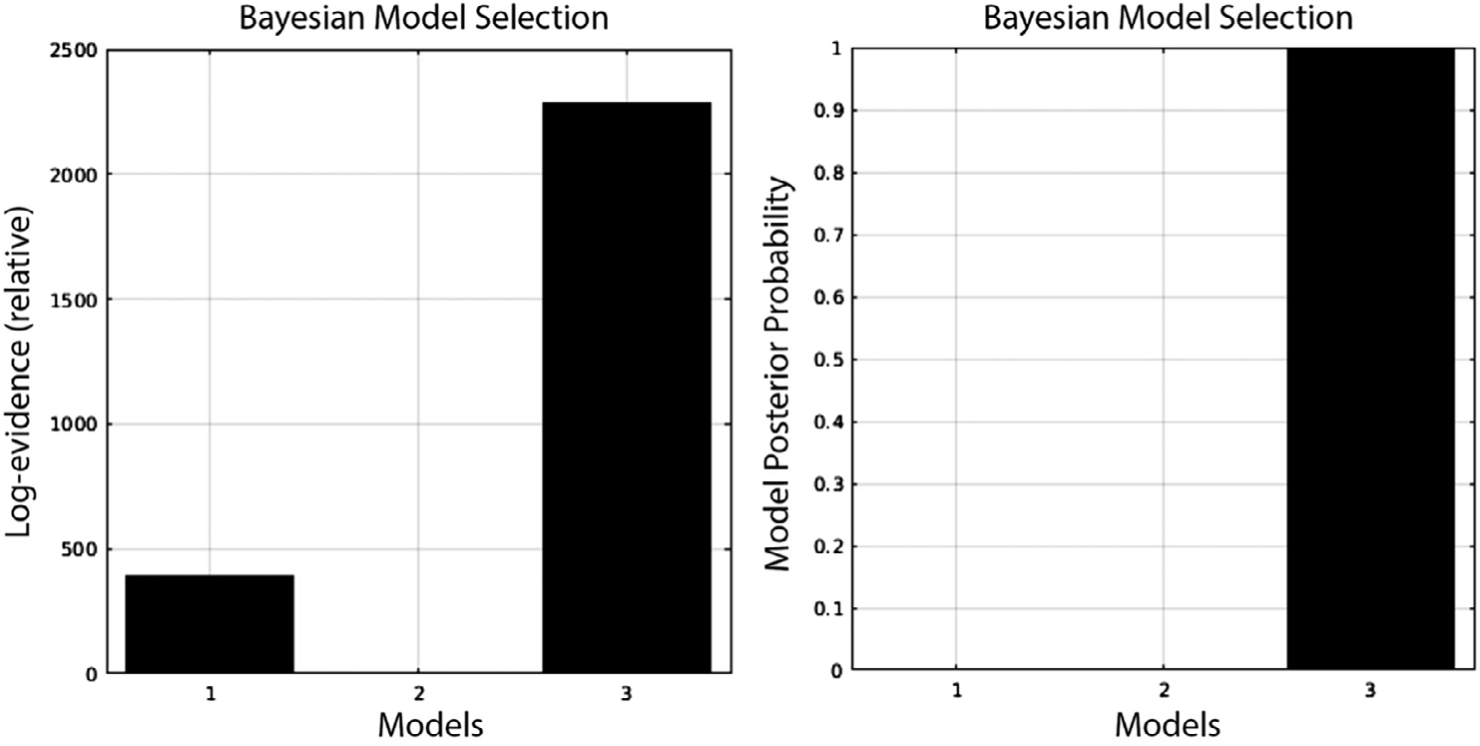
Results of comparison of three models, differing only in location of driving input. Model 1 = driving input to frontal only; Model 2 = driving input to parietal only; Model 3 = driving input to both frontal and parietal regions. Left graph: log evidence results; right graph: posterior probability results

In order to focus on the modulation of connectivity—between the frontal and parietal regions—without a potential influence of where the driving input was delivered, we allowed the driving input to enter each of the four regions. To test whether this choice was justified, we performed an initial DCM comparison, in which models varied in terms of which areas received driving input (only frontal vs. only parietal vs. both parietal and frontal). As can be seen in Figure 3, the model with driving inputs to both regions was clearly preferred over the other two models. Thus, in all subsequent analyses, the driving input entered all regions.

### 3.3 DCM: Bayesian model selection

**Figure 4:**
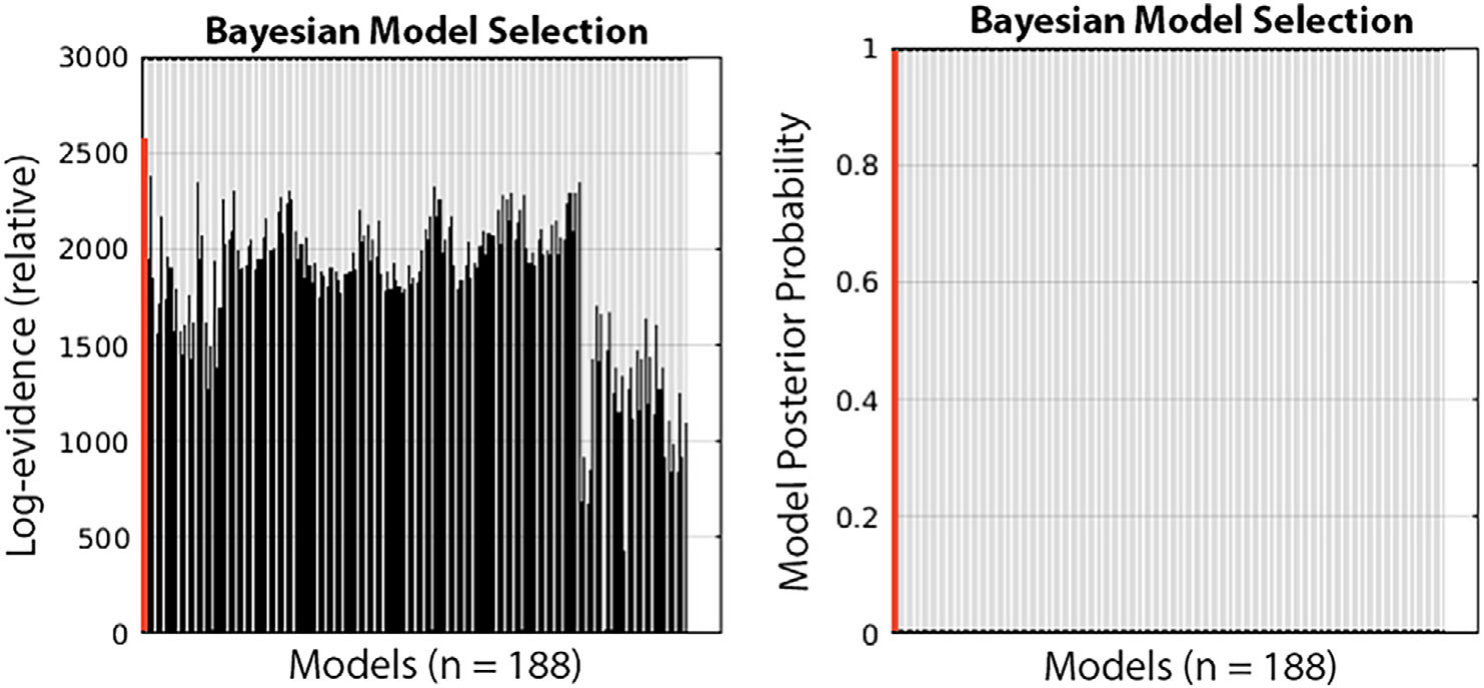
Relative model evidences for all 188 models tested against a baseline of the least‐probable model. Left graph: log evidence results; right graph: posterior probability results. The winning model (Model #1: full model with all frontal–parietal connections modulated by each attention type) is highlighted in red, at far left edge of each graph. Models are listed across the x axis in the order they were created; a full description of each of the 188 models is provided in Table S1 in the supplemental materials

Fixed‐effects BMS performed on our full *n* = 188 model set revealed a winning model (Figure 4), with a posterior probability of >99.9%, in which each of the frontoparietal connections, in both directions and in both hemispheres, are modulated by *each* of our three attention conditions (such as shown in Figure 2). It should be noted that DCM assigns a *lower* prior probability to models with more free parameters (e.g., more specified modulations), so this result was interesting in that it suggested that each of the attentional effects in our “full” model were necessary to explain the data above and beyond the inherent complexity cost of including an extra attentional parameter. As noted above, the winning model had a posterior probability of >99.9% in comparison to all other models; therefore, no other models received closely comparable support.

Regarding the large disparity in posterior probability between the winning model and all other models, this is partially due to our use of fixed‐effects Bayesian model selection, which is highly efficient because one has very precise estimates from each subject. If the estimates of model parameters are precise, the evidence for marginal likelihood is usually large, relative to the next best model. When plotted in terms of probability, this makes it look as if all the other models have a zero posterior probability. Indeed, DCM studies using fixed‐effects model selection have found posterior probabilities exceeding 99% for a single model, illustrating the utility of this selection scheme in scenarios such as ours where the fixed‐effects assumptions hold (Campo et al., 2012; Gibson et al., 2017; Vossel, Mathys, Stephan, & Friston, 2015). In these cases, as in ours, plots of log evidence (showing the relative evidence for each model on a logarithmic scale) can differentiate among all models considered (see also Supplemental Table S1). Note, however, that the current result of one clear winning model is not simply a generic behavior of all fixed‐effects DCM analyses; other studies have resulted in multiple models having high, sometimes equally plausible, posterior probabilities (Amini et al., 2018; Kellermann et al., 2012; Matsuyoshi et al., 2015; Murta, Leal, Garrido, & Figueiredo, 2012).

A full description of the architecture of all 188 models tested and their respective relative log evidences and posterior probabilities is included in the supplementary materials (Supplemental Table S1). All models had common intrinsic connectivity and driving input; models varied only in their modulatory influence on frontoparietal connections. With respect to model evidences, note that: (a) comparative evidence values are on a logarithmic scale, so differences in evidence between models are larger than the numerical metric may indicate; (b) SPM12 uses the calculated free energy of a DCM as an approximation to model evidence; and (c) evidence values are relativized to the model with the least evidence (i.e., that model's evidence is subtracted from all models).

In order to further investigate whether any of the 12 modulatory inputs in particular may be least critical, we compared the top 10 models with the highest log evidence (Supplemental Table S2). Comparing across these models, there was no consistency across the other top models in terms of which modulatory effect may not be necessary to explain the data. No modulatory effect was removed from more than 2 of the top 10 winning models, and none of the top 10 models eliminated more than two modulatory effects. Therefore, even if we consider the top *nonwinning* models, there is little evidence to suggest that a particular modulatory effect is redundant. Thus, in addition to the strong posterior probability of the winning, fully modulated model, inspection of other models that had high log evidence does not provide reason to suggest that any of the modulatory effects should be discounted.

### 3.4 DCM: Winning model parameters

**Figure 5:**
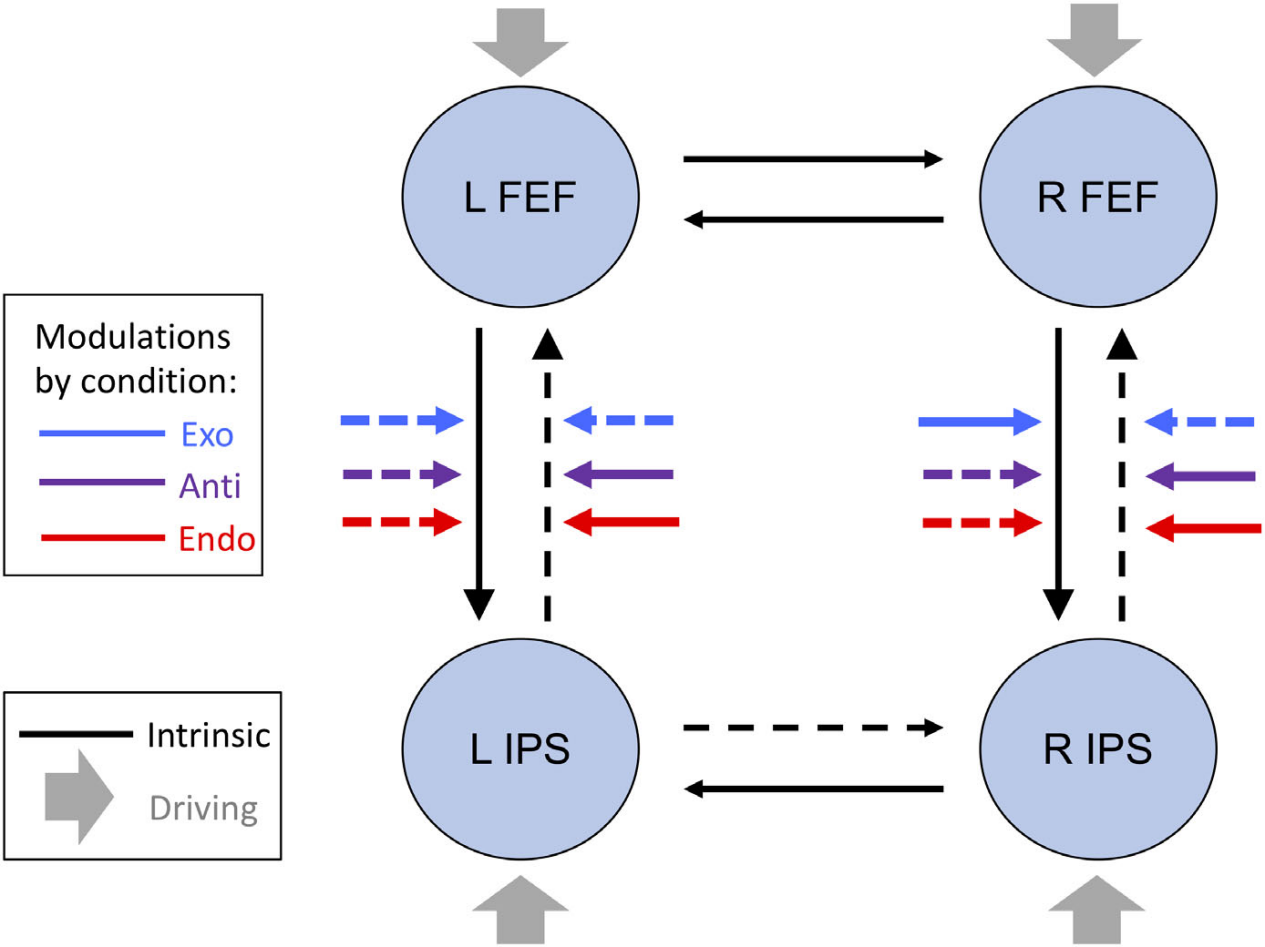
Winning model. Solid lines indicate positive parameter values (after Bayesian parameter averaging across subjects); dashed lines indicate effects with negative parameter values. Modulation of frontal–parietal connectivity by the Exo attention condition is seen to be the opposite (positive vs. negative) of the Endo and Anti modulations in all cases except for the frontal eye field (FEF)‐to‐parietal connection in the left hemisphere. Values of connections and modulations are provided in Tables 2 and 3

Having identified the winning model, we then quantified the connections and modulations under that model. Specifically, we analyzed the values of the coupling parameters in DCM, which represent the directed influence among neuronal populations (Cardin, Friston, & Zeki, 2011). Parameters of the connection (DCM.A matrix) are interpreted as the strength of one region's facilitatory (or inhibitory) effect on another, averaged over time, while the parameters of modulatory inputs (DCM.B, e.g., attentional effects) represent the change around the average connectivity between regions induced by experimental events. Using the most likely model structure, we performed BPA across the 19 participants. The winning model is displayed in Figure 5, and the parameter estimates under this model are reported in Tables 2 and 3. Posterior probabilities of these parameters being nonzero (DCM.Pp matrix) were all >99.9% (i.e., their posterior distributions did not overlap with 0). In other words, we can be nearly 100% certain that the Bayesian parameter averages were greater than or less than zero.

**Table 2:** Parameter values for intrinsic (average) connections (DCM.A) between ROIs in the winning model after averaging across subjects (unit: 1/s)

Connection	Intrinsic strength	Connection	Intrinsic strength
Left FEF to Left IPS	0.3578	Right FEF to Right IPS	0.8891
Left IPS to Left FEF	−1.2039	Right IPS to Right FEF	−0.2037
Left FEF to Right FEF	0.1281	Right FEF to Left FEF	0.8351
Left IPS to Right IPS	−1.0405	Right IPS to Left IPS	0.0722

Abbreviations: FEF, frontal eye field; IPS, intraparietal sulcus; ROI, region of interest.

**Table 3:** Parameter values for modulatory inputs (DCM.B) in the winning model after averaging across subjects (unit: 1/s)

Connection	Effect of Exo	Effect of Endo	Effect of Anti
Left FEF to Left IPS	−0.189	−0.2958	−0.6486
Right FEF to Right IPS	0.5572	−1.6863	−0.0942
Left IPS to Left FEF	−0.872	0.3363	0.824
Right IPS to Right FEF	−1.5809	0.8168	1.8157

Abbreviations: FEF, frontal eye field; IPS, intraparietal sulcus.

Our specific interest was in how frontal–parietal connections were differentially modified by the three attention conditions. As shown in Figure 5 and Table 3, the crucial connections between frontal and parietal areas were affected in distinct ways (i.e., strengthened vs. weakened) when comparing these different types of attention. These results provide new evidence that endogenous and exogenous attention have opposite effects on the majority of these connections. Specifically, for directed connectivity from IPS to FEF, exogenous attention increases an inhibitory connection (by adding negative influence), whereas endogenous attention has the opposite effect (reducing the inhibitory influence of parietal on frontal regions). The connection from FEF to IPS in the right hemisphere also shows an antisymmetric pattern of modulation for the two types of attention. Interestingly, the *anti‐predictive* condition has the *same* qualitative modulatory influence (positive vs. negative) as the *endogenous* condition on *all four* frontal–parietal connections. Since the physical stimulus is identical for the *exogenous* and anti‐predictive conditions, this suggests that the present results are not overly influenced by physical stimuli, but rather reflect the type of attention engaged. Together, these results provide evidence that the modulatory effects of purely exogenous attention were indeed distinct from the modulatory effects produced by endogenous attention.

In regards to hemispheric differences, it is noteworthy that the connection from FEF to IPS in the left hemisphere is modulated in the same direction by all types of attention tested here, whereas the FEF to IPS connection in the right hemisphere is modulated in an antisymmetric fashion for Endo versus Exo attention. This relates to our previous report, using this dataset, showing hemispheric asymmetry and further supports research suggesting that attention mechanisms may not be the same in the two hemispheres.

## 4 DISCUSSION

Much previous research has shown that a dorsal attention network is associated with attentional control. In the current study, we asked whether the directed connectivity between the frontal and parietal portions of this network is affected in different ways by endogenous versus exogenous attention. Using DCM, we assessed directed connectivity between the FEF and IPS, and how attention, across three different conditions, modulated that connectivity. Of 188 models tested, the winning model comprised modulations by each of our three attention conditions—on both directions of frontoparietal connectivity and within both hemispheres. This suggests that each type of attention had a substantial effect on frontal‐to‐parietal and parietal‐to‐frontal connectivity. Analysis of the parameters in the winning model, averaged across subjects, revealed a baseline (average) connectivity with a pattern of an inhibitory influence of parietal areas on frontal areas and a facilitatory effect of frontal areas on parietal areas. Crucially, for our main hypothesis, a distinct pattern of modulatory effects was observed for endogenous and exogenous attention on those frontoparietal connections. With the exception of the modulation of the FEF to IPS in the left hemisphere, exogenous attention modulated connectivity in the opposite direction as endogenous attention. Thus, these results provide new evidence for distinct neural mechanisms underlying exogenous versus endogenous attention.

From the perspective of predictive coding, this is very sensible. Most formulations of attention under predictive coding focus on the neuronal excitability of prediction error units that encode the precision of ascending (forward) prediction errors (Auksztulewicz & Friston, 2015; Feldman & Friston, 2010; Parr & Friston, 2019). Usually, the functional anatomy of attention under predictive coding models considers the early visual cortex as a source of ascending prediction errors (Bauer, Stenner, Friston, & Dolan, 2014; Feldman & Friston, 2010; Harrison, Stephan, Rees, & Friston, 2007; Kok, Rahnev, Jehee, Lau, & de Lange, 2012; Limanowski & Friston, 2018; Spratling, 2008). In the current setting, the particular results pertaining to attentional modulation of forward and backward connections fit comfortably when treating the FEF as the lower level in a simple two‐level hierarchy. This is sensible from several perspectives: first, the FEF represents lower level information that is prescient for attentional deployment: cf., the premotor theory of attention (Rizzolatti, Riggio, Dascola, & Umilta, 1987; Vossel et al., 2015; Wurtz, 2008). Furthermore, anatomical tracing studies suggest that FEF can occupy a relatively low level in the visual hierarchy, based upon asymmetries in the laminar specificity of extrinsic connections (Anderson, Kennedy, & Martin, 2011; Vezoli et al., 2004). Finally, detailed analysis of the microcircuitry in the motor cortex leads to the conclusion that (motor) executive components of sensorimotor hierarchies are hierarchically subordinate to regions such as the IPS (Shipp, 2016; Shipp, Adams, & Friston, 2013). On this view, the excitatory forward connections from FEF to IPS are consistent with the driving influences of ascending prediction errors (Lee & Mumford, 2003; Mumford, 1992; Rao & Ballard, 1999). Similarly, the inhibitory descending connections from IPS to FEF would correspond to the suppression of prediction errors at lower levels (Kersten, Mamassian, & Yuille, 2004; Murray, Kersten, Olshausen, Schrater, & Woods, 2002). Crucially, the effects of endogenous and exogenous attention can be interpreted as an endogenous inhibition of ascending prediction errors from FEF; with a complementary augmentation of (the inhibitory influence of) descending predictions from IPS during the deployment of exogenous attention (see Figure 5). Clearly, there are lots of simplifying assumptions that attend this interpretation; however, the overall pattern of effective connectivity—and its attentional modulation—yields to a straightforward predictive coding explanation.

Our finding—that the pattern of modulatory effects of these two types of attention was different in the right versus left hemisphere—also provides new evidence that the hemispheres are not identical in regards to these attentional processes. Recent results (Meehan et al., 2017) have underlined the importance of hemispheric differences in the frontoparietal network. Indeed, our previous analyses of these data (Meyer et al., 2018) found that the right hemisphere did not show differences in the balance of overall activity for FEF versus IPS, whereas the left hemisphere did. However, those previous analyses were only looking at simple activation levels in those regions separately. Here, by looking at distributed responses and directed connectivity among regions, we were able to disclose a crucial difference in how the dorsal attention network is modulated by endogenous versus exogenous attention. In this regard, the hemispheric differences between these studies may be especially informative. Whereas neuropsychological patient studies often suggest a right hemisphere dominance for spatial attention (e.g., Heilman & Van Den Abell, 1980; Mesulam, 1999; Posner, Walker, Friedrich, & Rafal, 1984), neuroimaging studies with healthy adults have reported equal or oftentimes greater activity in the left than right hemisphere within the dorsal frontoparietal network (Hopfinger et al., 2000; Kastner, Pinsk, De Weerd, Desimone, & Ungerleider, 1999; Shulman et al., 2010; Sommer, Kraft, Schmidt, Olma, & Brandt, 2008). Indeed, our previous analyses of these data (Meyer et al., 2018) found that the largest differences in overall responses during endogenous versus exogenous attention were located in the left hemisphere. This result in some respects raises the question: to what extent were the current hemispheric differences in modulation driven by the hemispheric asymmetry in BOLD activity found in the prior analysis? While further studies would be needed to definitively answer this question, it is unlikely that this calls into question the generalizability of our results, given that left‐hemispheric equivalence or preeminence has been observed repeatedly in activation‐based neuroimaging analyses of healthy adult populations (Hopfinger et al., 2000; Kastner et al., 1999; Shulman et al., 2010; Sommer et al., 2008). Furthermore, the current study highlights that when looking at the *directed* causal influences across regions, instead of simply activation levels, it is clear that the major *differences* in attentional *modulation* are located in the right hemisphere. The findings of hemispheric differences, in activity and modulatory influences of different types of attention, have important implications for studies, such as single‐unit recordings or neurostimulation, that typically investigate only a single hemisphere. It appears that, at least in our subjects, there are crucial hemispheric differences that should be accounted for to fully understand the mediation of bottom‐up and top‐down mechanisms of attention.

In addition to the exogenous and endogenous conditions, we also included an anti‐predictive condition that combined elements of both. By using the identical physical stimuli (as in the exogenous condition), the anti‐predictive condition allowed us to examine whether the pattern of modulations was dominated by the sensory input alone. The results showed, however, that the voluntary allocation of attention had a stronger impact on connectivity in this condition. Indeed, the direction of modulatory effect on all four frontal–parietal connections for the anti‐predictive condition was the same as for the endogenous condition. Although the anti‐predictive condition would be expected to show an initial triggering of the exogenous system, the present results confirm that predictive information—about the target location—has a robust and lasting effect on the allocation of attention and related neural dynamics.

Our DCM results show that top‐down attention, both when engaged via the endogenous central cue and via the anti‐predictive peripheral cue, consistently modulated connectivity in a way that *opposed* the observed intrinsic connectivity between frontal and parietal regions. This pattern is consistent with top‐down attention being an effortful control over perceptual and cognitive processes. In contrast, exogenous attention alone (in the nonpredictive peripheral cue) modulated connectivity in the manner of *boosting* intrinsic connectivity. This was consistent across the frontal–parietal connections, except in one case: exogenous attention modulated extrinsic frontal‐to‐parietal connectivity in the left hemisphere in a manner opposing the direction of opposing the intrinsic connectivity at that connection. Furthermore, at this one connection (left hemisphere frontal‐to‐parietal), exogenous and endogenous attention (triggered by either the predictive central cue or the anti‐predictive peripheral cue) modulated the connectivity in the same direction. The similarity in this specific modulatory effect across attention types was not predicted; however, it could potentially relate to the asymmetries observed in studies of attentional neglect. It has recently been suggested that endogenous orienting mechanisms remain intact in those with neglect, but the exogenous orienting system is disturbed (Karnath, 2015). Combined with the higher prevalence of neglect following right hemisphere versus left hemisphere damage (Molenberghs, Sale, & Mattingley, 2012), the current results may indicate a critical aspect for a healthy bottom‐up orienting system is the modulatory effect, whereby the exogenous attention system enhances connectivity from FEF to IPS in the right hemisphere, in direct opposition to the modulatory influence of top‐down attention. It is unclear why the direction of this modulation by exogenous attention would be in the opposite direction in the left hemisphere; however, since the parameter value (i.e., magnitude) for that modulatory effect is much smaller than the other exogenous modulatory effects, future studies may seek to resolve the strength and relative importance of that modulation compared to the other modulatory influences of exogenous attention.

Conclusions from the present study—about the functional anatomy of endogenous versus exogenous attention—rest on Bayesian model comparison. This means the evidence for different computational architectures of attentional set is limited to the particular paradigm we have used and to the subgraph considered in the DCM analyses (i.e., two regions in each hemisphere). Although these data were elicited under a carefully designed paradigm—and the regions were chosen in a principled way—our results should not be generalized to other subgraphs or experimental manipulations of attentional set. As with many DCM studies of computational architectures, the results in this article should be seen as contributing to the process of hypothesis testing and subsequent hypothesis building. In other words, certain questions now arise that call for further studies. For example, to what extent are the hemispheric differences in attention‐modulated connectivity seen here due to left hemispheric dominance? Would attention to different features—crossed with spatial attention—lead to similar results? The analyses in this article suggest that these sorts of questions can now be addressed and answered.

The current dataset was restricted to dorsal cortical regions including the dorsal frontoparietal network, which has been strongly implicated in attentional control through many neuropsychological patient studies (Friedrich et al., 1998; Henik et al., 1994; Mesulam, 1999) and neuroimaging studies in healthy adults (Corbetta et al., 1993; Corbetta et al., 2000; Hopfinger et al., 2000; Kastner et al., 1999; Nobre et al., 1997). An important direction for future studies would be to include other regions involved in attentional control networks. While DCM analyses typically include a relatively small number (<8) of carefully considered regions (in part owing to the exponential increase in computational load with each node added), recent work using constrained priors on coupling among modes suggests that analysis of larger graph DCMs could now be viable and could represent an important advance for future experiments (Razi et al., 2017; Seghier & Friston, 2013). In regards to understanding the dynamics of attentional control, it would be helpful to include the TPJ and ventral frontal cortex, as these regions have also been consistently implicated in unilateral neglect (see Karnath & Rorden, 2012 for review) and in reorienting in healthy participants (Corbetta, Patel, & Shulman, 2008; but see DiQuattro, Sawaki, & Geng, 2014). Our current finding of a hemispheric asymmetry in *modulatory effects* within the dorsal frontoparietal network, somewhat contrasts a prior finding of an absence of hemispheric asymmetry within the dorsal network during attentional shifts and target detection (Shulman et al., 2010); however, that study did find a right‐sided dominance of the TPJ. Since the TPJ often shows this pattern of hemispheric asymmetry (Kincade et al., 2005), adding that region into dynamic models such as those tested here could help explain the asymmetries we find when examining the differences between modulatory effects of these types of attention. In addition, including visual processing regions was shown to be helpful in a previous study that used Granger causality to reveal a right‐hemispheric asymmetry predictive of behavioral performance when analyzing connectivity between the FEF, IPS, and visual regions (Bressler, Tang, Sylvester, Shulman, & Corbetta, 2008). As the analysis of larger graph DCM methods is advanced, adding to the models in these ways should prove informative.

The interpretation of effective connectivity at the level of synaptic mechanisms has certain limits. The kind of neural mass models used in DCM of fMRI do not have the level of detail that distinguishes between different neurons or neuronal populations found in equivalent models for EEG (Brown & Friston, 2012). For example, in standard DCM for fMRI excitatory and inhibitory neurons are pooled into a single population. In turn, this means the interpretation of the polarity (i.e., negative and positive) of effective connectivity must be qualified. First, effective connectivity is polysynaptic; in other words, the effect of one neuronal population on another can be mediated monosynaptically or via an ensemble of indirect (postsynaptic) pathways. This means that different combinations of excitatory and inhibitory populations can switch the polarity of the effective connectivity. A key example of this is negative extrinsic (between regions) cortical connectivity, which is mediated by glutamatergic neurotransmission. This means that a negative directed (effective) connection has to be mediated by inhibitory neurons, which are usually the target of backward or descending extrinsic connections (Shipp, 2016). Similar arguments pertain to changes in connectivity. A particular example here is the hemispheric asymmetry between the effect of exogenous attention on the FEF to IPS connection, which is inhibitory for the left hemisphere but facilitatory for the right. This does not necessarily imply that different synaptic connections have been engaged under different attentional sets; it more likely reflects the differential modulation of intrinsic (within region) connectivity between excitatory (e.g., pyramidal) cells and inhibitory interneurons. This neuromodulation can have many mechanisms; ranging from changes in synchronous gain due to fast (oscillatory) neuronal dynamics to the modulation (via NMDA receptor agonism) of fast spiking GABAergic inhibitory interneurons—and their recurrent exchange with excitatory pyramidal cells (Anenberg, Chan, Xie, LeDue, & Murphy, 2015; Bauer, Oostenveld, Peeters, & Fries, 2006; Bosman et al., 2012; Breakspear, Heitmann, & Daffertshofer, 2010; Chawla, Rees, & Friston, 1999; Fries, Womelsdorf, Oostenveld, & Desimone, 2008; Lawrence, 2008; Lee, Whittington, & Kopell, 2013). Please see Daunizeau, David, and Stephan (2011) for a fuller discussion of the assumptions and qualifications that attend the interpretation of DCM studies. Furthermore, the ability of any method that utilizes correlational data is limited in terms of assessing causality. Neurostimulation methods, such as transcranial magnetic stimulation, transcranial direct current stimulation (tDCS), and transcranial alternating current stimulation, permit the exciting possibility to directly test the models and causal relations identified through neuroimaging studies (Blankenburg et al., 2010; Hopfinger et al., 2017; Moos, Vossel, Weidner, Sparing, & Fink, 2012; Ronconi, Basso, Gori, & Facoetti, 2012; Roy, Sparing, Fink, & Hesse, 2015).

Finally, recent work has investigated the frequency‐specific oscillatory activity that relates to different attentional control processes. This research has revealed that the alpha (8–12 Hz) and gamma frequency bands (30–100 Hz) may be especially important for understanding the mechanisms of attention. Changes in alpha activity are usually found ~500 ms after the instructive cue and are thought to be related to the biasing of sensory processing regions following a shift of attention (Thut, Nietzel, Brandt, & Pascual‐Leone, 2006; Yamagishi, Goda, Callan, Anderson, & Kawato, 2005). Other studies have revealed a burst of activity in the gamma band that precedes the alpha suppression (Vidal, Chaumon, O'Regan, & Tallon‐Baudry, 2006). Fan et al. (2007) further specified that gamma‐band activity at ~200 ms following the instructive cue is uniquely related to the orienting of attention (as opposed to alerting or executive control). This role in the orienting process has been further specified to reflect an endogenous shifting of attention, based on the finding that it is induced by an instructive peripheral cue but not by the same cue when it is uninformative (Landau, Esterman, Robertson, Bentin, & Prinzmetal, 2007). However, it remains unknown if this gamma burst occurs when attention shifts are not associated with an external cue stimulus. Nonhuman primate studies suggest that voluntary attention may rely more on low frequency oscillations, whereas involuntary attention may rely more on high spatial frequencies, which might coordinate local connections (Engel, Fries, & Singer, 2001) by mechanisms of synchronous gain. Indeed, in Buschman and Miller's (2007) study, the frontal to parietal flow of information was reflected at lower frequency ranges, whereas the parietal to frontal direction triggered by exogenous attention was reflected activity in higher spatial frequencies. The current study provides a first step in establishing that frontal–parietal directed connectivity is affected differentially by the type of attention being engaged; future studies using electrophysiological and neurostimulation measures in humans should investigate whether these differences are supported by neural activity at different frequencies, with accompanying spectral asymmetries (Bastos et al., 2015).

## CONFLICT OF INTEREST

All authors report no conflicts of interest.

## Supporting information


**Table S1** Architecture of all 188 models tested.

**Table S2** Top 10 models ranked on log evidence

## Data Availability

Data sharing statement The data that support the findings of this study are available from the corresponding author upon reasonable request.
