# Supplementary material for: Top‐down versus bottom‐up attention differentially modulate frontal–parietal connectivity
Source: Hum Brain Mapp. 2019 Nov 6;41(4):928–42. doi: 10.1002/hbm.24850 (PMC7267915; doi:10.1002/hbm.24850)
Supplement: Supplementary file 1 — Table S1 Architecture of all 188 models tested. [file HBM-41-928-s001.docx]

**Table S1: Architecture of all 188 models tested.**

Included modulatory inputs indicated by 'X' in table.

|  |  |  | **L IPS to**  **L FEF** |  |  |  | **L FEF to**  **L IPS** |  |  |  | **R IPS to**  **R FEF** |  |  |  | **R FEF to**  **R IPS** |  |  | **Log-evidence (relative)** | **Posterior Probability** |
| --- | --- | --- | --- | --- | --- | --- | --- | --- | --- | --- | --- | --- | --- | --- | --- | --- | --- | --- | --- |
| **Model** |  | **Endo** | **Exo** | **Anti** |  | **Endo** | **Exo** | **Anti** |  | **Endo** | **Exo** | **Anti** |  | **Endo** | **Exo** | **Anti** |  |  |  |
| **1** |  | X | X | X |  | X | X | X |  | X | X | X |  | X | X | X |  | 2570 | > 99.9% |
| **2** |  | X | X |  |  | X | X | X |  | X | X |  |  | X | X | X |  | 1950 | 0 |
| **3** |  | X | X | X |  | X | X |  |  | X | X | X |  | X | X |  |  | 2380 | 0 |
| **4** |  | X |  | X |  | X | X | X |  | X |  | X |  | X |  | X |  | 1850 | 0 |
| **5** |  | X |  |  |  | X | X | X |  | X |  |  |  | X | X | X |  | 1560 | 0 |
| **6** |  | X |  | X |  | X | X |  |  | X |  | X |  | X | X |  |  | 1720 | 0 |
| **7** |  | X | X | X |  | X |  | X |  | X | X | X |  | X |  | X |  | 2180 | 0 |
| **8** |  | X | X |  |  | X |  | X |  | X | X |  |  | X |  | X |  | 1740 | 0 |
| **9** |  | X | X | X |  | X |  |  |  | X | X | X |  | X |  |  |  | 1960 | 0 |
| **10** |  |  | X | X |  | X | X | X |  |  | X | X |  | X | X | X |  | 1910 | 0 |
| **11** |  |  | X |  |  | X | X | X |  |  | X |  |  | X | X | X |  | 1580 | 0 |
| **12** |  |  | X | X |  | X | X |  |  |  | X | X |  | X | X |  |  | 1800 | 0 |
| **13** |  |  |  | X |  | X | X | X |  |  |  | X |  | X | X | X |  | 1580 | 0 |
| **14** |  |  |  |  |  | X | X | X |  |  |  |  |  | X | X | X |  | 1460 | 0 |
| **15** |  |  |  | X |  | X | X |  |  |  |  | X |  | X | X |  |  | 1610 | 0 |
| **16** |  |  | X | X |  | X |  | X |  |  | X | X |  | X |  | X |  | 1770 | 0 |
| **17** |  |  | X |  |  | X |  | X |  |  | X |  |  | X |  | X |  | 1430 | 0 |
| **18** |  |  | X | X |  | X |  |  |  |  | X | X |  | X |  |  |  | 1620 | 0 |
| **19** |  | X | X | X |  |  | X | X |  | X | X | X |  |  | X | X |  | 2350 | 0 |
| **20** |  | X | X |  |  |  | X | X |  | X | X |  |  |  | X | X |  | 1950 | 0 |
| **21** |  | X | X | X |  |  | X |  |  | X | X | X |  |  | X |  |  | 2080 | 0 |
| **22** |  | X |  | X |  |  | X | X |  | X |  | X |  |  | X | X |  | 1620 | 0 |
| **23** |  | X |  |  |  |  | X | X |  | X |  |  |  |  | X | X |  | 1280 | 0 |
| **24** |  | X |  | X |  |  | X |  |  | X |  | X |  |  | X |  |  | 1500 | 0 |
| **25** |  | X | X | X |  |  |  | X |  | X | X | X |  |  |  | X |  | 1940 | 0 |
|  |  |  |  |  |  |  |  |  |  |  |  |  |  |  |  |  |  |  |  |
|  |  |  | **L IPS to**  **L FEF** |  |  |  | **L FEF to**  **L IPS** |  |  |  | **R IPS to**  **R FEF** |  |  |  | **R FEF to**  **R IPS** |  |  | **Log-evidence (relative)** | **Posterior Probability** |
| **Model** |  | **Endo** | **Exo** | **Anti** |  | **Endo** | **Exo** | **Anti** |  | **Endo** | **Exo** | **Anti** |  | **Endo** | **Exo** | **Anti** |  |  |  |
| **26** |  | X | X |  |  |  |  | X |  | X | X |  |  |  |  | X |  | 1390 | 0 |
| **27** |  | X | X | X |  |  |  |  |  | X | X | X |  |  |  |  |  | 1690 | 0 |
| **28** |  | X | X | X |  |  |  |  |  | X | X | X |  | X | X | X |  | 2260 | 0 |
| **29** |  | X | X |  |  |  |  | X |  | X | X | X |  | X | X | X |  | 2030 | 0 |
| **30** |  | X | X | X |  |  |  | X |  | X | X | X |  | X | X |  |  | 2050 | 0 |
| **31** |  | X | X | X |  |  |  | X |  | X | X |  |  | X | X | X |  | 2100 | 0 |
| **32** |  | X | X | X |  |  |  | X |  | X | X | X |  | X | X | X |  | 2300 | 0 |
| **33** |  | X |  | X |  |  | X |  |  | X | X | X |  | X | X | X |  | 1990 | 0 |
| **34** |  | X |  |  |  |  | X | X |  | X | X | X |  | X | X | X |  | 1900 | 0 |
| **35** |  | X |  | X |  |  | X | X |  | X | X | X |  | X | X |  |  | 1910 | 0 |
| **36** |  | X |  | X |  |  | X | X |  | X | X |  |  | X | X | X |  | 1920 | 0 |
| **37** |  | X |  | X |  |  | X | X |  | X | X | X |  | X | X | X |  | 2020 | 0 |
| **38** |  | X | X | X |  |  | X |  |  | X | X | X |  | X |  | X |  | 2050 | 0 |
| **39** |  | X | X |  |  |  | X | X |  | X | X | X |  | X |  | X |  | 1890 | 0 |
| **40** |  | X | X | X |  |  | X | X |  | X | X | X |  | X |  |  |  | 1960 | 0 |
| **41** |  | X | X | X |  |  | X | X |  | X | X |  |  | X |  | X |  | 1950 | 0 |
| **42** |  | X | X | X |  |  | X | X |  | X | X | X |  | X |  | X |  | 2060 | 0 |
| **43** |  | X | X | X |  |  | X |  |  | X |  | X |  | X | X | X |  | 2160 | 0 |
| **44** |  | X | X |  |  |  | X | X |  | X |  | X |  | X | X | X |  | 2000 | 0 |
| **45** |  | X | X | X |  |  | X | X |  | X |  | X |  | X | X |  |  | 2000 | 0 |
| **46** |  | X | X | X |  |  | X | X |  | X |  |  |  | X | X | X |  | 2010 | 0 |
| **47** |  | X | X | X |  |  | X | X |  | X |  | X |  | X | X | X |  | 2190 | 0 |
| **48** |  | X | X | X |  |  | X |  |  | X | X | X |  | X | X | X |  | 2270 | 0 |
| **49** |  | X | X |  |  |  | X | X |  | X | X | X |  | X | X | X |  | 2080 | 0 |
| **50** |  | X | X | X |  |  | X | X |  | X | X | X |  | X | X |  |  | 2240 | 0 |
| **51** |  | X | X | X |  |  | X | X |  | X | X |  |  | X | X | X |  | 2300 | 0 |
| **52** |  | X | X | X |  |  | X | X |  | X | X | X |  | X | X | X |  | 2270 | 0 |
| **53** |  |  | X | X |  | X |  |  |  | X | X | X |  | X | X | X |  | 2100 | 0 |
| **54** |  |  | X |  |  | X |  | X |  | X | X | X |  | X | X | X |  | 1950 | 0 |
| **55** |  |  | X | X |  | X |  | X |  | X | X | X |  | X | X |  |  | 2030 | 0 |
|  |  |  | **L IPS to**  **L FEF** |  |  |  | **L FEF to**  **L IPS** |  |  |  | **R IPS to**  **R FEF** |  |  |  | **R FEF to**  **R IPS** |  |  | **Log-evidence (relative)** | **Posterior Probability** |
| **Model** |  | **Endo** | **Exo** | **Anti** |  | **Endo** | **Exo** | **Anti** |  | **Endo** | **Exo** | **Anti** |  | **Endo** | **Exo** | **Anti** |  |  |  |
| **56** |  |  | X | X |  | X |  | X |  | X | X |  |  | X | X | X |  | 1860 | 0 |
| **57** |  |  | X | X |  | X |  | X |  | X | X | X |  | X | X | X |  | 2060 | 0 |
| **58** |  |  |  | X |  | X | X |  |  | X | X | X |  | X | X | X |  | 1920 | 0 |
| **59** |  |  |  |  |  | X | X | X |  | X | X | X |  | X | X | X |  | 1830 | 0 |
| **60** |  |  |  | X |  | X | X | X |  | X | X | X |  | X | X |  |  | 1930 | 0 |
| **61** |  |  |  | X |  | X | X | X |  | X | X |  |  | X | X | X |  | 1750 | 0 |
| **62** |  |  |  | X |  | X | X | X |  | X | X | X |  | X | X | X |  | 1880 | 0 |
| **63** |  |  | X | X |  | X | X |  |  | X | X | X |  | X |  | X |  | 1860 | 0 |
| **64** |  |  | X |  |  | X | X | X |  | X | X | X |  | X |  | X |  | 1800 | 0 |
| **65** |  |  | X | X |  | X | X | X |  | X | X | X |  | X |  |  |  | 1910 | 0 |
| **66** |  |  | X | X |  | X | X | X |  | X | X |  |  | X |  | X |  | 1910 | 0 |
| **67** |  |  | X | X |  | X | X | X |  | X | X | X |  | X |  | X |  | 1880 | 0 |
| **68** |  |  | X | X |  | X | X |  |  | X |  | X |  | X | X | X |  | 1840 | 0 |
| **69** |  |  | X |  |  | X | X | X |  | X |  | X |  | X | X | X |  | 1770 | 0 |
| **70** |  |  | X | X |  | X | X | X |  | X |  | X |  | X | X |  |  | 1880 | 0 |
| **71** |  |  | X | X |  | X | X | X |  | X |  |  |  | X | X | X |  | 1870 | 0 |
| **72** |  |  | X | X |  | X | X | X |  | X |  | X |  | X | X | X |  | 1890 | 0 |
| **73** |  |  | X | X |  | X | X |  |  | X | X | X |  | X | X | X |  | 1980 | 0 |
| **74** |  |  | X |  |  | X | X | X |  | X | X | X |  | X | X | X |  | 1900 | 0 |
| **75** |  |  | X | X |  | X | X | X |  | X | X | X |  | X | X |  |  | 2210 | 0 |
| **76** |  |  | X | X |  | X | X | X |  | X | X |  |  | X | X | X |  | 2040 | 0 |
| **77** |  |  | X | X |  | X | X | X |  | X | X | X |  | X | X | X |  | 2070 | 0 |
| **78** |  | X | X | X |  | X |  |  |  | X | X | X |  |  | X | X |  | 2120 | 0 |
| **79** |  | X | X |  |  | X |  | X |  | X | X | X |  |  | X | X |  | 1940 | 0 |
| **80** |  | X | X | X |  | X |  | X |  | X | X | X |  |  | X |  |  | 2050 | 0 |
| **81** |  | X | X | X |  | X |  | X |  | X | X |  |  |  | X | X |  | 1960 | 0 |
| **82** |  | X | X | X |  | X |  | X |  | X | X | X |  |  | X | X |  | 2150 | 0 |
| **83** |  | X |  | X |  | X | X |  |  | X | X | X |  |  | X | X |  | 1870 | 0 |
| **84** |  | X |  |  |  | X | X | X |  | X | X | X |  |  | X | X |  | 1780 | 0 |
| **85** |  | X |  | X |  | X | X | X |  | X | X | X |  |  | X |  |  | 1880 | 0 |
|  |  |  | **L IPS to**  **L FEF** |  |  |  | **L FEF to**  **L IPS** |  |  |  | **R IPS to**  **R FEF** |  |  |  | **R FEF to**  **R IPS** |  |  | **Log-evidence (relative)** | **Posterior Probability** |
| **Model** |  | **Endo** | **Exo** | **Anti** |  | **Endo** | **Exo** | **Anti** |  | **Endo** | **Exo** | **Anti** |  | **Endo** | **Exo** | **Anti** |  |  |  |
| **86** |  | X |  | X |  | X | X | X |  | X | X |  |  |  | X | X |  | 1800 | 0 |
| **87** |  | X |  | X |  | X | X | X |  | X | X | X |  |  | X | X |  | 1930 | 0 |
| **88** |  | X | X | X |  | X | X |  |  | X | X | X |  |  |  | X |  | 1840 | 0 |
| **89** |  | X | X |  |  | X | X | X |  | X | X | X |  |  |  | X |  | 1810 | 0 |
| **90** |  | X | X | X |  | X | X | X |  | X | X | X |  |  |  |  |  | 1780 | 0 |
| **91** |  | X | X | X |  | X | X | X |  | X | X |  |  |  |  | X |  | 1800 | 0 |
| **92** |  | X | X | X |  | X | X | X |  | X | X | X |  |  |  | X |  | 1920 | 0 |
| **93** |  | X | X | X |  | X | X |  |  | X |  | X |  |  | X | X |  | 1820 | 0 |
| **94** |  | X | X |  |  | X | X | X |  | X |  | X |  |  | X | X |  | 1850 | 0 |
| **95** |  | X | X | X |  | X | X | X |  | X |  | X |  |  | X |  |  | 1830 | 0 |
| **96** |  | X | X | X |  | X | X | X |  | X |  |  |  |  | X | X |  | 1890 | 0 |
| **97** |  | X | X | X |  | X | X | X |  | X |  | X |  |  | X | X |  | 2000 | 0 |
| **98** |  | X | X | X |  | X | X |  |  | X | X | X |  |  | X | X |  | 2100 | 0 |
| **99** |  | X | X |  |  | X | X | X |  | X | X | X |  |  | X | X |  | 2060 | 0 |
| **100** |  | X | X | X |  | X | X | X |  | X | X | X |  |  | X |  |  | 2170 | 0 |
| **101** |  | X | X | X |  | X | X | X |  | X | X |  |  |  | X | X |  | 2330 | 0 |
| **102** |  | X | X | X |  | X | X | X |  | X | X | X |  |  | X | X |  | 2170 | 0 |
| **103** |  | X | X | X |  | X |  |  |  |  | X | X |  | X | X | X |  | 2260 | 0 |
| **104** |  | X | X |  |  | X |  | X |  |  | X | X |  | X | X | X |  | 1990 | 0 |
| **105** |  | X | X | X |  | X |  | X |  |  | X | X |  | X | X |  |  | 2050 | 0 |
| **106** |  | X | X | X |  | X |  | X |  |  | X |  |  | X | X | X |  | 2120 | 0 |
| **107** |  | X | X | X |  | X |  | X |  |  | X | X |  | X | X | X |  | 2180 | 0 |
| **108** |  | X |  | X |  | X | X |  |  |  | X | X |  | X | X | X |  | 1920 | 0 |
| **109** |  | X |  |  |  | X | X | X |  |  | X | X |  | X | X | X |  | 1790 | 0 |
| **110** |  | X |  | X |  | X | X | X |  |  | X | X |  | X | X |  |  | 1840 | 0 |
| **111** |  | X |  | X |  | X | X | X |  |  | X |  |  | X | X | X |  | 1840 | 0 |
| **112** |  | X |  | X |  | X | X | X |  |  | X | X |  | X | X | X |  | 1920 | 0 |
| **113** |  | X | X | X |  | X | X |  |  |  | X | X |  | X |  | X |  | 2040 | 0 |
| **114** |  | X | X |  |  | X | X | X |  |  | X | X |  | X |  | X |  | 1850 | 0 |
| **115** |  | X | X | X |  | X | X | X |  |  | X | X |  | X |  |  |  | 1930 | 0 |
|  |  |  | **L IPS to**  **L FEF** |  |  |  | **L FEF to**  **L IPS** |  |  |  | **R IPS to**  **R FEF** |  |  |  | **R FEF to**  **R IPS** |  |  | **Log-evidence (relative)** | **Posterior Probability** |
| **Model** |  | **Endo** | **Exo** | **Anti** |  | **Endo** | **Exo** | **Anti** |  | **Endo** | **Exo** | **Anti** |  | **Endo** | **Exo** | **Anti** |  |  |  |
| **116** |  | X | X | X |  | X | X | X |  |  | X |  |  | X |  | X |  | 1910 | 0 |
| **117** |  | X | X | X |  | X | X | X |  |  | X | X |  | X |  | X |  | 2020 | 0 |
| **118** |  | X | X | X |  | X | X |  |  |  |  | X |  | X | X | X |  | 2100 | 0 |
| **119** |  | X | X |  |  | X | X | X |  |  |  | X |  | X | X | X |  | 1970 | 0 |
| **120** |  | X | X | X |  | X | X | X |  |  |  | X |  | X | X |  |  | 2080 | 0 |
| **121** |  | X | X | X |  | X | X | X |  |  |  |  |  | X | X | X |  | 2080 | 0 |
| **122** |  | X | X | X |  | X | X | X |  |  |  | X |  | X | X | X |  | 2070 | 0 |
| **123** |  | X | X | X |  | X | X |  |  |  | X | X |  | X | X | X |  | 2200 | 0 |
| **124** |  | X | X |  |  | X | X | X |  |  | X | X |  | X | X | X |  | 2030 | 0 |
| **125** |  | X | X | X |  | X | X | X |  |  | X | X |  | X | X |  |  | 2280 | 0 |
| **126** |  | X | X | X |  | X | X | X |  |  | X |  |  | X | X | X |  | 2260 | 0 |
| **127** |  | X | X | X |  | X | X | X |  |  | X | X |  | X | X | X |  | 2150 | 0 |
| **128** |  | X | X | X |  | X |  |  |  | X | X | X |  | X | X | X |  | 2290 | 0 |
| **129** |  | X | X |  |  | X |  | X |  | X | X | X |  | X | X | X |  | 2050 | 0 |
| **130** |  | X | X | X |  | X |  | X |  | X | X | X |  | X | X |  |  | 2140 | 0 |
| **131** |  | X | X | X |  | X |  | X |  | X | X |  |  | X | X | X |  | 2210 | 0 |
| **132** |  | X | X | X |  | X |  | X |  | X | X | X |  | X | X | X |  | 2280 | 0 |
| **133** |  | X |  | X |  | X | X |  |  | X | X | X |  | X | X | X |  | 2010 | 0 |
| **134** |  | X |  |  |  | X | X | X |  | X | X | X |  | X | X | X |  | 1930 | 0 |
| **135** |  | X |  | X |  | X | X | X |  | X | X | X |  | X | X |  |  | 1980 | 0 |
| **136** |  | X |  | X |  | X | X | X |  | X | X |  |  | X | X | X |  | 1920 | 0 |
| **137** |  | X |  | X |  | X | X | X |  | X | X | X |  | X | X | X |  | 2050 | 0 |
| **138** |  | X | X | X |  | X | X |  |  | X | X | X |  | X |  | X |  | 2110 | 0 |
| **139** |  | X | X |  |  | X | X | X |  | X | X | X |  | X |  | X |  | 1970 | 0 |
| **140** |  | X | X | X |  | X | X | X |  | X | X | X |  | X |  |  |  | 2000 | 0 |
| **141** |  | X | X | X |  | X | X | X |  | X | X |  |  | X |  | X |  | 1970 | 0 |
| **142** |  | X | X | X |  | X | X | X |  | X | X | X |  | X |  | X |  | 2130 | 0 |
| **143** |  | X | X | X |  | X | X |  |  | X |  | X |  | X | X | X |  | 2150 | 0 |
| **144** |  | X | X |  |  | X | X | X |  | X |  | X |  | X | X | X |  | 1970 | 0 |
| **145** |  | X | X | X |  | X | X | X |  | X |  | X |  | X | X |  |  | 2060 | 0 |
|  |  |  | **L IPS to**  **L FEF** |  |  |  | **L FEF to**  **L IPS** |  |  |  | **R IPS to**  **R FEF** |  |  |  | **R FEF to**  **R IPS** |  |  | **Log-evidence (relative)** | **Posterior Probability** |
| **Model** |  | **Endo** | **Exo** | **Anti** |  | **Endo** | **Exo** | **Anti** |  | **Endo** | **Exo** | **Anti** |  | **Endo** | **Exo** | **Anti** |  |  |  |
| **146** |  | X | X | X |  | X | X | X |  | X |  |  |  | X | X | X |  | 2050 | 0 |
| **147** |  | X | X | X |  | X | X | X |  | X |  | X |  | X | X | X |  | 2240 | 0 |
| **148** |  | X | X | X |  | X | X |  |  | X |  | X |  | X | X | X |  | 2300 | 0 |
| **149** |  | X | X |  |  | X | X | X |  | X |  | X |  | X | X | X |  | 2100 | 0 |
| **150** |  | X | X | X |  | X | X | X |  | X |  | X |  | X | X |  |  | 2300 | 0 |
| **151** |  | X | X | X |  | X | X | X |  | X |  |  |  | X | X | X |  | 2350 | 0 |
| **152** |  |  |  |  |  |  |  |  |  |  |  |  |  |  |  |  |  | 690 | 0 |
| **153** |  |  |  | X |  |  |  | X |  |  |  | X |  |  |  | X |  | 930 | 0 |
| **154** |  |  |  |  |  |  |  | X |  |  |  |  |  |  |  | X |  | 680 | 0 |
| **155** |  |  |  | X |  |  |  |  |  |  |  | X |  |  |  |  |  | 860 | 0 |
| **156** |  | X |  |  |  | X |  |  |  | X |  |  |  | X |  |  |  | 1430 | 0 |
| **157** |  | X |  | X |  | X |  | X |  | X |  | X |  | X |  | X |  | 1700 | 0 |
| **158** |  | X |  |  |  | X |  | X |  | X |  |  |  | X |  | X |  | 1420 | 0 |
| **159** |  | X |  | X |  | X |  |  |  | X |  | X |  | X |  |  |  | 1670 | 0 |
| **160** |  | X | X |  |  | X | X |  |  | X | X |  |  | X | X |  |  | 0 | 0 |
| **161** |  | X |  |  |  | X | X |  |  | X |  |  |  | X | X |  |  | 1480 | 0 |
| **162** |  | X | X |  |  | X |  |  |  | X | X |  |  | X |  |  |  | 1680 | 0 |
| **163** |  |  |  |  |  | X |  |  |  |  |  |  |  | X |  |  |  | 1260 | 0 |
| **164** |  |  |  | X |  | X |  | X |  |  |  | X |  | X |  | X |  | 1390 | 0 |
| **165** |  |  |  |  |  | X |  | X |  |  |  |  |  | X |  | X |  | 1150 | 0 |
| **166** |  |  |  | X |  | X |  |  |  |  |  | X |  | X |  |  |  | 1350 | 0 |
| **167** |  |  | X |  |  | X | X |  |  |  | X |  |  | X | X |  |  | 440 | 0 |
| **168** |  |  |  |  |  | X | X |  |  |  |  |  |  | X | X |  |  | 1270 | 0 |
| **169** |  |  | X |  |  | X |  |  |  |  | X |  |  | X |  |  |  | 1390 | 0 |
| **170** |  | X |  |  |  |  |  |  |  | X |  |  |  |  |  |  |  | 1130 | 0 |
| **171** |  | X |  | X |  |  |  | X |  | X |  | X |  |  |  | X |  | 1480 | 0 |
| **172** |  | X |  |  |  |  |  | X |  | X |  |  |  |  |  | X |  | 1170 | 0 |
| **173** |  | X |  | X |  |  |  |  |  | X |  | X |  |  |  |  |  | 1430 | 0 |
| **174** |  | X | X |  |  |  | X |  |  | X | X |  |  |  | X |  |  | 1650 | 0 |
| **175** |  | X |  |  |  |  | X |  |  | X |  |  |  |  | X |  |  | 1200 | 0 |
|  |  |  | **L IPS to**  **L FEF** |  |  |  | **L FEF to**  **L IPS** |  |  |  | **R IPS to**  **R FEF** |  |  |  | **R FEF to**  **R IPS** |  |  | **Log-evidence (relative)** | **Posterior Probability** |
| **Model** |  | **Endo** | **Exo** | **Anti** |  | **Endo** | **Exo** | **Anti** |  | **Endo** | **Exo** | **Anti** |  | **Endo** | **Exo** | **Anti** |  |  |  |
| **176** |  | X | X |  |  |  |  |  |  | X | X |  |  |  |  |  |  | 1440 | 0 |
| **177** |  |  | X |  |  |  | X |  |  |  | X |  |  |  | X |  |  | 1150 | 0 |
| **178** |  |  | X | X |  |  | X | X |  |  | X | X |  |  | X | X |  | 1610 | 0 |
| **179** |  |  | X |  |  |  | X | X |  |  | X |  |  |  | X | X |  | 1280 | 0 |
| **180** |  |  | X | X |  |  | X |  |  |  | X | X |  |  | X |  |  | 1380 | 0 |
| **181** |  |  |  |  |  |  | X |  |  |  |  |  |  |  | X |  |  | 920 | 0 |
| **182** |  |  |  | X |  |  | X | X |  |  |  | X |  |  | X | X |  | 1110 | 0 |
| **183** |  |  |  |  |  |  | X | X |  |  |  |  |  |  | X | X |  | 850 | 0 |
| **184** |  |  |  | X |  |  | X |  |  |  |  | X |  |  | X |  |  | 980 | 0 |
| **185** |  |  | X |  |  |  |  |  |  |  | X |  |  |  |  |  |  | 850 | 0 |
| **186** |  |  | X | X |  |  |  | X |  |  | X | X |  |  |  | X |  | 1260 | 0 |
| **187** |  |  | X |  |  |  |  | X |  |  | X |  |  |  |  | X |  | 920 | 0 |
| **188** |  |  | X | X |  |  |  |  |  |  | X | X |  |  |  |  |  | 1100 | 0 |
