# Supplementary material for: Top‐down versus bottom‐up attention differentially modulate frontal–parietal connectivity
Source: Hum Brain Mapp. 2019 Nov 6;41(4):928–42. doi: 10.1002/hbm.24850 (PMC7267915; doi:10.1002/hbm.24850)
Supplement: Supplementary file 2 — Table S2 Top 10 models ranked on log evidence [file HBM-41-928-s002.docx]

**Table S2: Top 10 models ranked on log-evidence**

Included modulatory connections indicated by 'x' in table.

**L IPS to L FEF L FEF to L IPS R IPS to R FEF R FEF to R IPS**

| **Model** |  | **Endo** | **Exo** | **Anti** |  | **Endo** | **Exo** | **Anti** |  | **Endo** | **Exo** | **Anti** |  | **Endo** | **Exo** | **Anti** |  | **(relative)** | **Probability** |
| --- | --- | --- | --- | --- | --- | --- | --- | --- | --- | --- | --- | --- | --- | --- | --- | --- | --- | --- | --- |
| **1** |  | X | X | X |  | X | X | X |  | X | X | X |  | X | X | X |  | 2570 | > 99.9% |
| **3** |  | X | X | X |  | X | X |  |  | X | X | X |  | X | X |  |  | 2380 | 0 |
| **19** |  | X | X | X |  |  | X | X |  | X | X | X |  |  | X | X |  | 2350 | 0 |
| **151** |  | X | X | X |  | X | X | X |  | X |  |  |  | X | X | X |  | 2350 | 0 |
| **101** |  | X | X | X |  | X | X | X |  | X | X |  |  |  | X | X |  | 2330 | 0 |
| **32** |  | X | X | X |  |  |  | X |  | X | X | X |  | X | X | X |  | 2300 | 0 |
| **51** |  | X | X | X |  |  | X | X |  | X | X |  |  | X | X | X |  | 2300 | 0 |
| **148** |  | X | X | X |  | X | X |  |  | X |  | X |  | X | X | X |  | 2300 | 0 |
| **150** |  | X | X | X |  | X | X | X |  | X |  | X |  | X | X |  |  | 2300 | 0 |
| **128** |  | X | X | X |  | X |  |  |  | X | X | X |  | X | X | X |  | 2290 | 0 |
